# Comparative and phylogenetic analyses of *Swertia* L. (Gentianaceae) medicinal plants (from Qinghai, China) based on complete chloroplast genomes

**DOI:** 10.1590/1678-4685-GMB-2021-0092

**Published:** 2021-12-13

**Authors:** Xin Xu, Jinping Li, Ran Chu, Mengjie Luan, Hongyu Wang, Kexin Song, Shixia Wei, Yuhua Shi, Shixin Zhu, Zhen Wei

**Affiliations:** 1 Zhengzhou University, School of Life Sciences, Zhengzhou, Henan, China.; 2 Qinghai Normal University, School of Life Sciences, Xining, Qinghai, China.; 3 Key Laboratory of Medicinal Animal and Plant Resources of Qinghai-Tibetan Plateau in Qinghai Province, Xining, Qinghai, China.

**Keywords:** Swertia, Zangyinchen, chloroplast genome, Gentianaceae, Qinghai-Tibet Plateau

## Abstract

*Swertia* L. is a large genus in Swertiinae (Gentianaceae). In China, many *Swertia* species are used as traditional Tibetan medicines, known as “Zangyinchen” or “Dida”. However, the phylogenetic relationships among *Swertia* medicinal plants and their wild relatives have remained unclear. In this study, we sequenced and assembled 16 complete chloroplast (cp) genomes of 10 *Swertia* species, mainly distributed in Qinghai Province, China. The results showed that these species have typical structures and characteristics of plant cp genomes. The sizes of *Swertia* cp genomes are ranging from 149,488 bp to 154,097 bp. Most *Swertia* cp genomes presented 134 genes, including 85 protein coding genes, eight rRNA genes, 37 tRNA genes, and four pseudogenes. Furthermore, the GC contents and boundaries of cp genomes are similar among *Swertia* species. The phylogenetic analyses indicated that *Swertia* is a complex polyphyletic group. In addition, positive selection was found in *psaI* and *petL* genes, indicating the possible adaptation of Qinghai *Swertia* species to the light environment of the Qinghai-Tibet plateau. These new cp genome data could be further investigated to develop DNA barcodes for *Swertia* medicinal plants and for additional systematic studies of *Swertia* and Swertiinae species.

*Swertia* L., a large genus of plants in Swertiinae (Gentianaceae) with about 170 species, are mainly distributed in Asia, Africa, North America and Europe (Ho *et al*., 1994). In China, around 79 *Swertia* species are mostly distributed in mountainous areas of the southwest, especially in the Himalayas (Ho and James, 1995; Joshi and Joshi, 2008). Some *Swertia* species are often used as Tibetan medicines, known as ‘Zangyinchen’ or ‘Dida’ to treat hepatobiliary diseases (Zhong *et al*., 2009). Although *Swertia* species have a long history of medicinal use, the systematics of the genus and regional groups remain obscure (von Hagen and Kadereit, 2001, 2002; Kadereit and von Hagen, 2003; Xi *et al*., 2014). In the present study, we assembled and annotated the complete chloroplast (cp) genomes of 16 *Swertia* taxa (10 *Swertia* species) mainly distributed in Qinghai province, China. The chloroplast genomes of these *Swertia* species were compared and used to study the phylogenetic relationships and selection pressure.

Fresh leaves of the plants were collected mainly in the Qinghai area (Table S1). Total genomic DNA was extracted using a modified CTAB method (Wei *et al*., 2017) and then purified with a Wizard^®^ DNA Clean-Up System (Promega, Beijing, China). The DNA samples were sequenced using Illumina HiSeq Xten Platforms. Cp genomes were assembled using NOVOPlasty v4.0 (Dierckxsens *et al*., 2016), annotated and manually corrected in Geneious Prime 2020.1.2 (Kearse *et al*., 2012). The structures and genes of the cp genomes were visualized by OrganellarGenomeDRAW (Lohse *et al*., 2013). All sequenced cp genomes were submitted to GenBank (Table S2).

All *Swertia* cp genomes have typical cp genome structures, including a large single copy region (LSC), a small single copy region (SSC), and two inverted repeat regions (IRs). A gene map of *Swertia bimaculata* Hook.f. & Thomson ex C.B. Clarke (MW344293) was shown as an example (Figure 1), and other new cp genomes are shown in supplementary figures (Figure S1-S9). The length, GC content, and gene number of 15 *Swertia* species and seven relatives in Swertiinae were determined and compared (Table S3). The total length of *Swertia* cp genomes range from 149,488 bp to 154,097 bp. The total GC content of the cp genomes are relatively stable (38.0%-38.2%), while the GC content of the IRs are higher than that of single copy regions. The cp genomes of other related species did not show any distinct differences from *Swertia* species. Most *Swertia* cp genomes contain 134 genes, including 85 protein coding genes, eight rRNA genes, 37 tRNA genes, and four pseudogenes (*rps16*, *infA*, *ycf1* and *rps19* genes) (Table S4). Gene deletion and additional pseudogenes were found in some *Swertia* cp genomes, as results of additional termination codons, complete or partial gene loss (Table S5). Previous studies have shown that some genes with defects or loss in cp genomes may have other copies with normal function in nuclear genomes, which could help avoid death or damage during plant growth (Martin *et al*., 1998; Sugiura *et al*., 2003).

**Figure 1 -:**
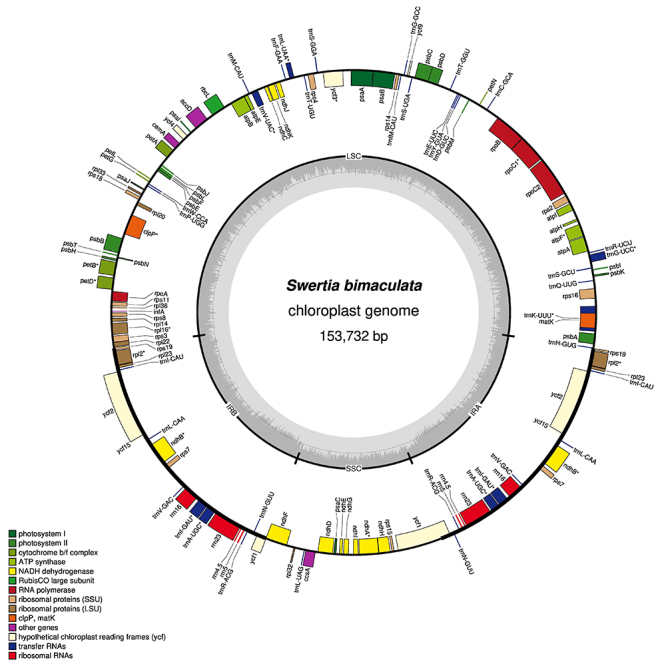
Gene map of the *Swertia bimaculata* (MW344293) chloroplast genome. The translation of genes outside the outer circle occurs in a counter-clockwise direction, while the translation of genes inside occurs in a clockwise direction. The dark and light gray colors in the inner circle represent GC and AT content, respectively. Different functional gene groups are highlighted by different colors.

The mVISTA software was used to evaluate the sequence conservations among genomes (https://genome.lbl.gov/vista/index.shtml). The structures and sequences of *Swertia* cp genomes are conserved, especially in the IR regions (Figure S10), probably due to the existence of rRNA genes and gene conversion (Khakhlova and Bock, 2006). Variation rates of coding and noncoding regions were calculated using Geneious Prime 2020.1.2. The results showed the variation rates of coding regions are generally lower than those of noncoding regions (Figure 2), and the variation rates of noncoding regions in the IR are lower than those in other regions. These regions of high variations could be used to develop new DNA barcodes of *Swertia* species and two pairs of newly designed primers were provided in the supplements (Table S6). Differences in the four boundaries of the LSC, SSC, and IRs were illustrated using the IRscope online website (https://irscope.shinyapps.io/irapp/). Boundary comparisons in *Swertia* species found that the structures of boundaries are conservative with slight differences among taxa (Figure 3). The boundaries of the four regions between *Swertia* and its relatives showed expansion and contraction to some extent.

**Figure 2 -:**
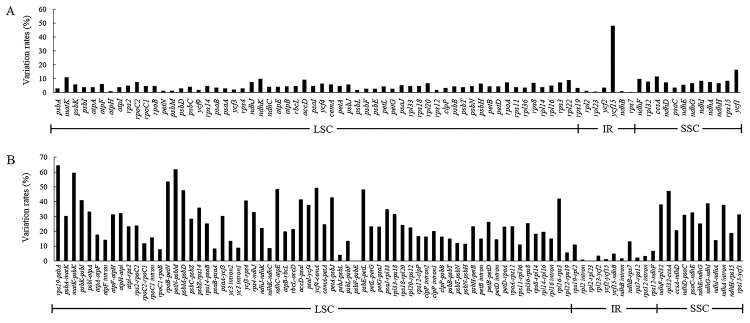
Variation rates of coding and noncoding regions in the chloroplast genomes of 15 *Swertia* species. A: Variation rates of 78 protein coding genes; the y-axis represents protein coding genes. B: Variation rates of noncoding regions; the y-axis represents intergenic spacers or introns. Abbreviations: LSC- large single copy region; SSC- small single copy region; IR- inverted repeat region.

**Figure 3 -:**
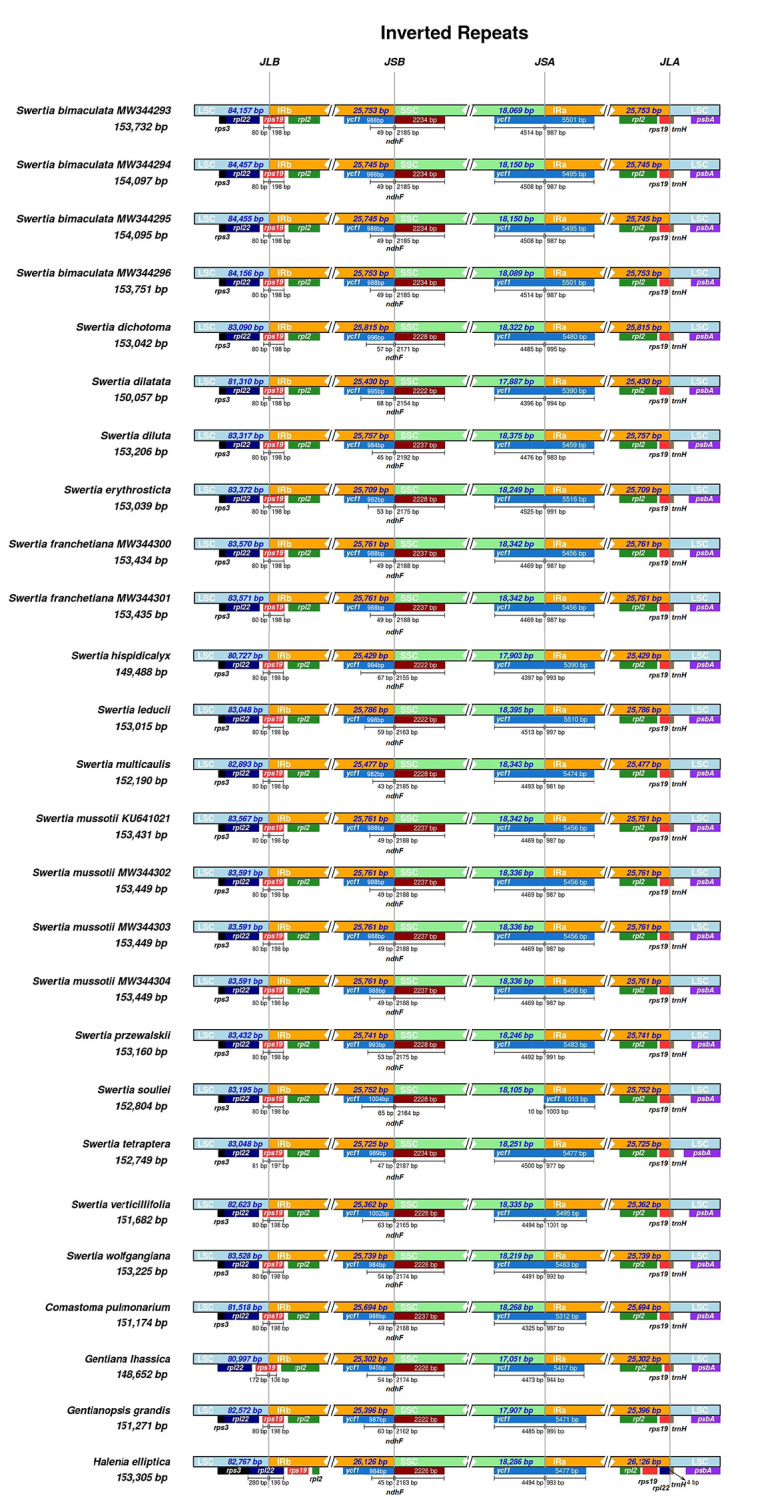
Boundaries of the LSC, SSC, and IRs in chloroplast genomes of 15 *Swertia* and four related species. LSC: large single copy region; SSC: small single copy region; IRa and IRb: two inverted repeat regions; JLB: junction between the LSC and IRb; JSB: junction between the SSC and IRb; JSA: junction between the SSC and IRa; JLA: junction between the LSC and IRa.

Phylogenetic trees were constructed based on 54 complete cp genomes of Gentianeae species and an Apocynaceae species as outgroup. Additional cp genomes used in the phylogenetic analysis were downloaded from the National Center for Biotechnology Information database (https://www.ncbi.nlm.nih.gov/) (Table S2). We applied Bayesian inference (BI), maximum likelihood (ML), and maximum parsimony (MP) methods to construct phylogenetic trees. The ML tree and BI tree were constructed in CIPRES Science Gateway (https://www.phylo.org/portal2/login!input.action) using RAxML-HPC2 on XSEDE and MrBayes on XSEDE. For ML tree, GTR+GAMMA was chosen as the model for the bootstrapping phase. The bootstrap replicates were 5000, and “print branch lengths (-k)” was chosen under “Configure Bootstrapping”. Other parameters were default settings. For BI analyses, the parameters were: lset nst = 6 rates = gamma; unlink statefreq = ( all ) revmat = ( all ) shape = ( all ) pinvar = ( all ); prset applyto = ( all ) ratepr = variable; mcmcp ngen = 5000000, relburnin = yes, burninfrac = 0.25, printfreq = 1000, samplefreq = 5000, nchains = 4 temp = 0.05. Other parameters were default settings. The MP tree was structured using PAUP v4.0a with bootstrap 3000 and default parameters. The output tree files were modified in TreeGraph 2 (http://treegraph.bioinfweb.info/) and Figtree v1.4.3 (http://tree.bio.ed.ac.uk/software/figtree/).

Phylogenetic trees based on three methods had the same topologies (Figure 4). All Gentianeae species formed two monophyletic clades: Gentianinae and Swertiinae. *Swertia* formed a large clade with *Comastoma* Toyokuni, *Halenia* Borkh., *Lomatogoniopsis* T.N. Ho & S.W. Liu, *Lomatogonium* A. Braun and *Veratrilla* Franch, indicating that *Swertia* is a complex polyphyletic group. *Swertia*, *Comastoma*, *Halenia*, *Lomatogoniopsis*, *Lomatogonium* and *Veratrilla* were traditionally thought to be independent taxonomic units based on morphology (Ho and James, 1995), though many of the species were used as ‘Zangyinchen’ or ‘Dida’ medicines to treat hepatobiliary diseases. Similar results of polyphyletic relationships were reported previously in studies of Swertiinae species based on fragments of cp and nuclear genomes. One possible explanation for such discrepancies is that morphological features are plastic (Kadereit and von Hagen, 2003); they may be affected by many factors, such as heredity and environment, and character changes may not always be revealed by molecular data. Additionally, *S. hispidicalyx* and *S. dilatata* are sister groups and their *rpl33* genes were completely lost in the cp genomes. This gene loss might have contributed to their close relationships in the phylogenetic tree. To maintain the stability of concept, *Swertia* species should be divided into smaller genera or merged with *Comastoma*, *Halenia*, *Lomatogoniopsis*, *Lomatogonium* and *Veratrilla* into larger genera. A combination of morphological data and nuclear genome data will be required in future research to resolve the phylogenetic relationships among genera in Swertiinae.

**Figure 4 -:**
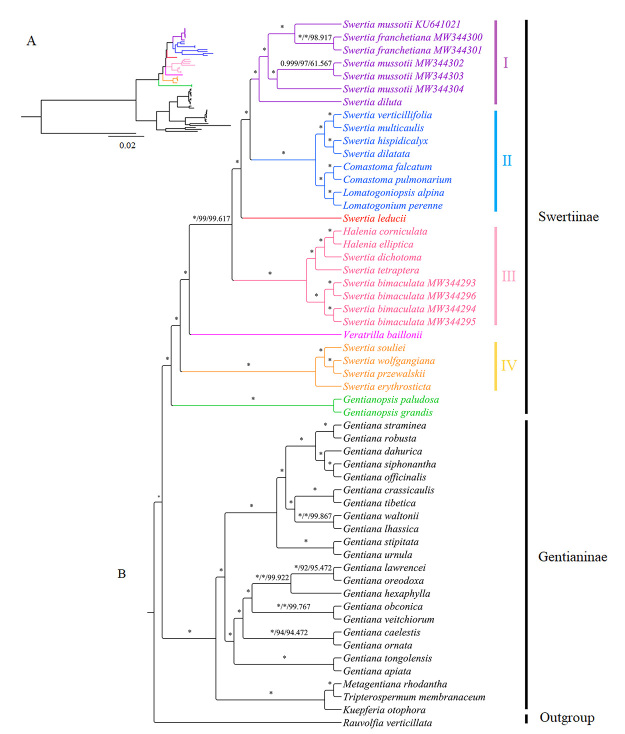
Phylogenetic tree of 54 Gentianeae species and one outgroup based on the complete chloroplast genomes. A: tree with branch lengths; B: tree without branch lengths. Supporting values are shown in the form of BI/ML/MP. The symbol * indicates full support. Abbreviations: BI: Bayesian inference; ML: maximum likelihood; MP: maximum parsimony.

Selection pressure was analyzed in Datamonkey (http://www.datamonkey.org/) using the Fixed Effects Likelihood Method. The values of non-synonymous/synonymous rate ratios (dn/ds) were calculated based on the 78 protein coding genes of 15 *Swertia* species, ranging from 0 to 1.65 (Figure S11). The ratios of *petL* (1.34) and *psaI* (1.65) were greater than one, meaning the two genes are under the influence of positive selection. Most genes with dn/ds values between zero and one are under purifying selection. A few genes have dn/ds values close to one, including *atpF*, *cemA*, *psbN*, *ycf2*, *ycf15*, and *ycf1* genes. These genes may be subject to weak positive selection. The *Swertia* plants studied here are mainly distributed across the Qinghai-Tibet plateau and other high-altitude areas, which are subject to large temperature differences between day and night, low overall temperatures, and high levels of ultraviolet radiation (Körner, 2003). When non-synonymous mutations can provide more survival opportunities for species, their rates can exceed that of synonymous mutations over a long period and be preserved in the population (Wolfe and ó’hUigín, 2016). The *psaI* gene codes for the *psaI* subunit of photosystem I (PSI) in plants and plays a role in the trimerization of PSI by stabilizing the combination of *psaL* to the light-harvesting complex (Plöchinger *et al*., 2016). The *petL* gene codes for subunits of the cytochrome *b6f* complex, involved in electron transport from photosystem II (PSII) to PSI (Schwenkert *et al*., 2007). The positive selection on *psaI* and *petL* genes may reflect the adaptation of *Swertia* to the light environment of the Qinghai-Tibet plateau.

Overall, we described here 16 newly sequenced cp genomes (10 species) of *Swertia*, and made comparative, phylogenetic and selection pressure analyses of them and other related species. Our results indicated that Qinghai *Swertia* species form a polyphyletic group and have similar structures and characteristics in cp genomes. Two genes were clearly under positive selection in *Swertia* cp genomes (*psaI* and *petL* genes), while some other genes were likely subject to weak selection. The results in this study could be utilized for developing DNA barcodes for medicinal *Swertia* plants and further phylogenetic study in *Swertia* and Swertiinae.

## Supplementary material

The following online material are available for this article:

Table S1 - Information of plant materials.

Table S2 - Accession numbers of the chloroplast genomes in this study.

Table S3 - Characteristics of chloroplast genomes from 15 *Swertia* species and seven related species.

Table S4 - Genes in *Swertia* chloroplast genomes.

Table S5 - The pseudogenes in *Swertia* chloroplast genomes.

Table S6 - Information of primer sequences.

Figure S1 - Gene maps of *S. dichotoma* chloroplast genome.

Figure S2 - Gene maps of *S. dilatata* chloroplast genome.

Figure S3 - Gene maps of *S. diluta* chloroplast genome.

Figure S4 - Gene maps of *S. erythrosticta* chloroplast genome.

Figure S5 - Gene maps of *S. franchetiana* chloroplast genome.

Figure S6 - Gene maps of *S. mussotii* chloroplast genome.

Figure S7 - Gene maps of *S. przewalskii* chloroplast genome.

Figure S8 - Gene maps of *S. tetraptera* chloroplast genome.

Figure S9 - Gene maps of *S. wolfgangiana* chloroplast genome.

Figure S10 - The alignment of chloroplast genomes for 15 *Swertia* species and four related species. *S. wolfgangiana* was used as a reference.

Figure S11 - Non-synonymous/synonymous rate ratios of the protein coding genes from the chloroplast genomes of 15 *Swertia* species.
