# Supplementary material for: Comparative and phylogenetic analyses of Swertia L. (Gentianaceae) medicinal plants (from Qinghai, China) based on complete chloroplast genomes
Source: Genet Mol Biol. 2021 Dec 13;45(1):e20210092. doi: 10.1590/1678-4685-GMB-2021-0092 (PMC8679245; doi:10.1590/1678-4685-GMB-2021-0092)
Supplement: Table S1 - [file 1415-4757-GMB-45-1-e20210092-s1.pdf]

Genetics and Molecular Biology
https://doi.org/10.1590/1678-4685-GMB-2021-0092

Supplementary Material to "Comparative and phylogenetic analyses of Swertia L. (Gentianaceae) medicinal plants (from Qinghai, China) based on complete chloroplast genomes"

Table S1 - Information of plant materials.

Species	Collection number	Collection location	Collector	Collection time
S. bimaculata MW344293	ZZU2019-6308	Luanchuan, Henan, China	S. X. Zhu	2019.8
S. bimaculata MW344294	ZZU2019-6312	Gongshan, Yunnan, China	Y. Lu	2019.10
S. bimaculata MW344295	ZZU2019-6313	Gongshan, Yunnan, China	Y. Lu	2019.10
S. bimaculata MW344296	ZZU2020-6314	Diebu, Gansu, China	Zeng *et al.*	2020.8
S. dichotoma	ZZU2020-6315	Xunhua, Qinghai, China	Zeng *et al.*	2020.8
S. dilatata	ZZU2020-6316	Langxian, Xizang, China	Zeng *et al.*	2020.8
S. diluta	ZZU2020-6318	Xixia, Henan, China	Y. Lu, H. Dong	2020.9
S. erythrosticta	ZZU2019-6309	Hualong, Qinghai, China	Zeng *et al.*	2019.8
S. franchetiana MW344300	ZZU2019-6310	Menyuan, Qinghai, China	Zeng *et al.*	2019.8
S. franchetiana MW344301	ZZU2019-6311	Hualong, Qinghai, China	Zeng *et al.*	2019.8
S. mussotii MW344302	ZZU2018-6301	Chengduo, Qinghai, China	Zeng *et al.*	2018.8
S. mussotii MW344303	ZZU2018-6302	Chengduo, Qinghai, China	Zeng *et al.*	2018.8
S. mussotii MW344304	ZZU2018-6303	Chengduo, Qinghai, China	Zeng *et al.*	2018.8
S. przewalskii	ZZU2020-6317	Menyuan, Qinghai, China	Zeng *et al.*	2020.8
S. tetraptera	ZZU2018-6304	Henan, Qinghai, China	Zeng *et al.*	2018.8
S. wolfgangiana	ZZU2019-6307	Maduo, Qinghai, China	Zeng *et al.*	2019.8
