# Supplementary material for: Comparative and phylogenetic analyses of Swertia L. (Gentianaceae) medicinal plants (from Qinghai, China) based on complete chloroplast genomes
Source: Genet Mol Biol. 2021 Dec 13;45(1):e20210092. doi: 10.1590/1678-4685-GMB-2021-0092 (PMC8679245; doi:10.1590/1678-4685-GMB-2021-0092)
Supplement: Table S2 - [file 1415-4757-GMB-45-1-e20210092-s2.pdf]

Genetics and Molecular Biology
https://doi.org/10.1590/1678-4685-GMB-2021-0092

Supplementary Material to "Comparative and phylogenetic analyses of Swertia L. (Gentianaceae) medicinal plants (from Qinghai, China) based on complete chloroplast genomes"

Table S2 - Accession numbers of the chloroplast genomes in this study.

Species	Taxonomy	GenBank accession number
Comastoma falcatum	Gentianaceae; Gentianeae; Swertiinae; Comastoma	MK331815
Comastoma pulmonarium	Gentianaceae; Gentianeae; Swertiinae; Comastoma	MT228723
Gentiana apiata	Gentianaceae; Gentianeae; Gentianinae; Gentiana	MK317975
Gentiana caelestis	Gentianaceae; Gentianeae; Gentianinae; Gentiana	MG192304
Gentiana ornata	Gentianaceae; Gentianeae; Gentianinae; Gentiana	MG192308
Gentiana hexaphylla	Gentianaceae; Gentianeae; Gentianinae; Gentiana	MG192305
Gentiana lawrencei	Gentianaceae; Gentianeae; Gentianinae; Gentiana	KX096882
Gentiana oreodoxa	Gentianaceae; Gentianeae; Gentianinae; Gentiana	MG192307
Gentiana obconica	Gentianaceae; Gentianeae; Gentianinae; Gentiana	MG192306
Gentiana veitchiorum	Gentianaceae; Gentianeae; Gentianinae; Gentiana	MG192310
Gentiana crassicaulis	Gentianaceae; Gentianeae; Gentianinae; Gentiana	KJ676538
Gentiana crassicaulis	Gentianaceae; Gentianeae; Gentianinae; Gentiana	KY595459
Gentiana tibetica	Gentianaceae; Gentianeae; Gentianinae; Gentiana	KU975374
Gentiana lhassica	Gentianaceae; Gentianeae; Gentianinae; Gentiana	MK790135
Gentiana waltonii	Gentianaceae; Gentianeae; Gentianinae; Gentiana	MK780032
Gentiana dahurica	Gentianaceae; Gentianeae; Gentianinae; Gentiana	MH261259
Gentiana officinalis	Gentianaceae; Gentianeae; Gentianinae; Gentiana	MH261261
Gentiana siphonantha	Gentianaceae; Gentianeae; Gentianinae; Gentiana	MH261260
Gentiana robusta	Gentianaceae; Gentianeae; Gentianinae; Gentiana	KT159969
Gentiana straminea	Gentianaceae; Gentianeae; Gentianinae; Gentiana	KJ657732
Gentiana stipitata	Gentianaceae; Gentianeae; Gentianinae; Gentiana	MG192309
Gentiana tongolensis	Gentianaceae; Gentianeae; Gentianinae; Gentiana	MK251985
Gentianopsis grandis	Gentianaceae; Gentianeae; Swertiinae; Gentianopsis	MT591268
Gentianopsis paludosa	Gentianaceae; Gentianeae; Swertiinae; Gentianopsis	MT228725
Halenia corniculata	Gentianaceae; Gentianeae; Swertiinae; Halenia	MK606372
Halenia elliptica	Gentianaceae; Gentianeae; Swertiinae; Halenia	MT228726
Kuepferia otophora	Gentianaceae; Gentianeae; Gentianinae; Kuepferia	MT228727
Lomatogoniopsis alpina	Gentianaceae; Gentianeae; Swertiinae; Lomatogoniopsis	MT228728

Species	Taxonomy	GenBank accession number
Lomatogonium perenne	Gentianaceae; Gentianeae; Swertiinae; Lomatogonium	MT228729
Metagentiana rhodantha	Gentianaceae; Gentianeae; Gentianinae; Metagentiana	MN822304
Rauvolfia verticillata	Apocynaceae; Rauvolfioideae; Vinceae; Rauvolfiinae; Rauvolfia	MN480804
*Swertia bimaculata**	Gentianaceae; Gentianeae; Swertiinae; Swertia	MW344293
*Swertia bimaculata**	Gentianaceae; Gentianeae; Swertiinae; Swertia	MW344294
*Swertia bimaculata**	Gentianaceae; Gentianeae; Swertiinae; Swertia	MW344295
*Swertia bimaculata**	Gentianaceae; Gentianeae; Swertiinae; Swertia	MW344296
*Swertia dichotoma**	Gentianaceae; Gentianeae; Swertiinae; Swertia	MW344297
*Swertia dilatata**	Gentianaceae; Gentianeae; Swertiinae; Swertia	MW344298
*Swertia diluta**	Gentianaceae; Gentianeae; Swertiinae; Swertia	MW338735
*Swertia erythrosticta**	Gentianaceae; Gentianeae; Swertiinae; Swertia	MW344299
*Swertia franchetiana**	Gentianaceae; Gentianeae; Swertiinae; Swertia	MW344300
*Swertia franchetiana**	Gentianaceae; Gentianeae; Swertiinae; Swertia	MW344301
Swertia hispidicalyx	Gentianaceae; Gentianeae; Swertiinae; Swertia	MH321887
Swertia leducii	Gentianaceae; Gentianeae; Swertiinae; Swertia	MN609998
Swertia multicaulis	Gentianaceae; Gentianeae; Swertiinae; Swertia	MT228730
*Swertia mussotii**	Gentianaceae; Gentianeae; Swertiinae; Swertia	MW344302
*Swertia mussotii**	Gentianaceae; Gentianeae; Swertiinae; Swertia	MW344303
*Swertia mussotii**	Gentianaceae; Gentianeae; Swertiinae; Swertia	MW344304
Swertia mussotii	Gentianaceae; Gentianeae; Swertiinae; Swertia	KU641021
*Swertia przewalskii**	Gentianaceae; Gentianeae; Swertiinae; Swertia	MW344305
Swertia souliei	Gentianaceae; Gentianeae; Swertiinae; Swertia	MT185926
*Swertia tetraptera**	Gentianaceae; Gentianeae; Swertiinae; Swertia	MW344306
Swertia verticillifolia	Gentianaceae; Gentianeae; Swertiinae; Swertia	MF795137
*Swertia wolfgangiana**	Gentianaceae; Gentianeae; Swertiinae; Swertia	MW344307
Tripterospermum membranaceum	Gentianaceae; Gentianeae; Gentianinae; Tripterospermum	MT228731
Veratrilla baillonii	Gentianaceae; Gentianeae; Swertiinae; Veratrilla	MT228732

* chloroplast genomes sequenced in this study.
