# Supplementary material for: Comparative and phylogenetic analyses of Swertia L. (Gentianaceae) medicinal plants (from Qinghai, China) based on complete chloroplast genomes
Source: Genet Mol Biol. 2021 Dec 13;45(1):e20210092. doi: 10.1590/1678-4685-GMB-2021-0092 (PMC8679245; doi:10.1590/1678-4685-GMB-2021-0092)
Supplement: Table S3 - [file 1415-4757-GMB-45-1-e20210092-s3.pdf]

Genetics and Molecular Biology
https://doi.org/10.1590/1678-4685-GMB-2021-0092

Supplementary Material to "Comparative and phylogenetic analyses of Swertia L. (Gentianaceae) medicinal plants (from Qinghai, China) based on complete chloroplast genomes"

Table S3 - Characteristics of chloroplast genomes from 15 *Swertia* species and seven related species.

Species	Length (bp)				GC content (%)				Number			
	Total	LSC	SSC	IR	Total	LSC	SSC	IR	Gene	CDS	rRNA	tRNA
S. bimaculata MW344293	153,732	84,157	18,069	25,753	38.0	36.0	32.1	43.4	134	85	8	37
S. bimaculata MW344294	154,097	84,457	18,150	25,745	38.0	36.0	32.0	43.4	134	85	8	37
S. bimaculata MW344295	154,095	84,456	18,149	25,745	38.0	36.0	32.0	43.4	134	85	8	37
S. bimaculata MW344296	153,751	84,156	18,089	25,753	38.0	36.0	32.1	43.4	134	85	8	37
S. dichotoma MW344297	153,042	83,090	18,322	25,815	38.1	36.2	31.9	43.4	134	85	8	37
S. dilatata MW344298	150,057	81,310	17,887	25,430	38.2	36.3	31.8	43.4	132	83	8	37
S. diluta MW338735	153,206	83,317	18,375	25,757	38.2	36.3	31.9	43.5	134	84	8	37
S. erythrosticta MW344299	153,039	83,372	18,249	25,709	38.1	36.2	31.9	43.4	134	85	8	37
S. franchetiana MW344300	153,434	83,570	18,342	25,761	38.2	36.2	31.9	43.5	134	85	8	37
S. franchetiana MW344301	153,435	83,571	18,342	25,761	38.2	36.2	31.9	43.5	134	85	8	37
S. hispidicalyx MH321887	149,488	80,727	17,903	25,429	38.2	36.3	31.8	43.4	132	83	8	37
S. leducii MN609998	153,015	83,048	18,395	25,786	38.2	36.3	31.9	43.3	134	85	8	37
S. multicaulis MT228730	152,190	82,893	18,343	25,477	38.1	36.3	31.8	43.4	134	85	8	37
S. mussotii KU641021	153,431	83,567	18,342	25,761	38.2	36.2	31.9	43.5	134	85	8	37
S. mussotii MW344302	153,449	83,591	18,336	25,761	38.2	36.2	31.9	43.5	134	85	8	37
S. mussotii MW344303	153,449	83,591	18,336	25,761	38.2	36.2	31.9	43.5	134	85	8	37
S. mussotii MW344304	153,449	83,591	18,336	25,761	38.2	36.2	31.9	43.5	134	85	8	37
S. przewalskii MW344305	153,160	83,432	18,246	25,741	38.1	36.2	31.9	43.3	134	85	8	37
S. souliei MT185926	152,804	83,195	18,105	25,752	38.1	36.2	31.9	43.3	134	85	8	37
S. tetraptera MW344306	152,742	83,047	18,237	25,729	38.1	36.2	31.9	43.4	134	85	8	37
S. verticillifolia MF795137	151,682	82,623	18,335	25,362	38.1	36.3	31.8	43.5	134	85	8	37
S. wolfgangiana MW344307	153,225	83,528	18,237	25,730	38.1	36.2	31.9	43.3	134	85	8	37
Comastoma pulmonarium MT228723	151,174	81,518	18,268	25,694	38.3	36.3	31.8	43.7	133	86	8	37
Gentiana lhassica MK790135	148,652	80,997	17,051	25,302	37.7	35.5	31.6	43.4	132	85	8	37
Gentianopsis grandis	151,271	82,572	17,907	25,396	37.9	35.8	31.8	43.4	133	85	8	37

Species	Length (bp)				GC content (%)				Number			
	Total	LSC	SSC	IR	Total	LSC	SSC	IR	Gene	CDS	rRNA	tRNA
MT591268												
Halenia elliptica MT228726	153,305	82,767	18,286	26,126	38.2	36.3	32.0	43.3	135	86	8	37
Lomatogoniopsis alpina MT228728	150,986	81,302	18,180	25,752	38.1	36.2	31.4	43.6	133	85	8	37
Lomatogonium perenne MT228729	151,678	81,979	18,237	25,731	38.2	36.3	31.5	43.6	133	85	8	37
Veratrilla baillonii MT228732	151,977	82,490	17,983	25,752	38.2	36.3	32.0	43.4	134	84	8	37
