# Supplementary material for: Comparative and phylogenetic analyses of Swertia L. (Gentianaceae) medicinal plants (from Qinghai, China) based on complete chloroplast genomes
Source: Genet Mol Biol. 2021 Dec 13;45(1):e20210092. doi: 10.1590/1678-4685-GMB-2021-0092 (PMC8679245; doi:10.1590/1678-4685-GMB-2021-0092)
Supplement: Table S4 - [file 1415-4757-GMB-45-1-e20210092-s4.pdf]

Genetics and Molecular Biology
https://doi.org/10.1590/1678-4685-GMB-2021-0092

Supplementary Material to "Comparative and phylogenetic analyses of Swertia L. (Gentianaceae) medicinal plants (from Qinghai, China) based on complete chloroplast genomes"

Table S4 - Genes in *Swertia* chloroplast genomes.

Category	Type of genes	Name of genes
Genes for Photosynthesis	Photosystem I	*psaA, psaB, psaC, psaI, psaJ*
	Photosystem II	*psbA, psbB, psbC, psbD, psbE, psbF, psbH, psbI, psbJ, psbK, psbL, psbM, psbN, psbT, psbZ*
	ATP synthase	*atpA, atpB, atpE, atpF, atpH, atpI*
	NADH dehydrogenase	*ndhA, ndhB*(2), *ndhC, ndhD, ndhE, ndhF, ndhG, ndhH, ndhI, ndhJ, ndhK*
	Cytochrome b_6f complex	*petA, petB, petD, petG, petL, petN*
	RuBisCO large subunit	*rbcL*
Genes for Self-replication	Small subunit ribosomal protein	*rps2, rps3, rps4, rps7*(2), *rps8, rps11, rps12, rps14, rps15, rps16*, rps18, rps19*, rps19*
	Large subunit Ribosomal protein	*rpl2*(2), *rpl14, rpl16, rpl20, rpl22, rpl23*(2), *rpl32, rpl33, rpl36*
	RNA polymerase	*rpoA, rpoB, rpoC1, rpoC2*
	Ribosomal RNA gene	*rrn 4.5*(2), *rrn5*(2), *rrn16*(2), *rrn23*(2)
	Transfer RNA gene	*trnA-UGC* (2), *trnC-GCA, trnD-GUC, trnE-UUC, trnF-GAA, trnfM-CAU, trnG-GCC, trnG-UCC, trnH-GUG, trnI-GAU* (2), *trnK-UUU, trnL-CAA* (2), *trnL-UAA, trnL-UAG, trnM-CAU* (3), *trnN-GUU* (2), *trnP-UGG, trnQ-UUG, trnR-ACG* (2), *trnR-UCU, trnS-GCU, trnS-GGA, trnS-UGA, trnT-GGU, trnT-UGU, trnV-GAC* (2), *trnV-UAC, trnW-CCA, trnY-GUA*
Other genes	Translational initiation factor IF-1	*infA**
	Maturase K	*matK*
	Acetyl-CoA carboxylase	*accD*
	Membrane protein	*cemA*
	Cytochrome c biogenesis	*ccsA*
	ATP-dependent protease	*clpP*
	hypothetical chloroplast open	*ycf1*, ycf1, ycf2*(2), *ycf3, ycf4, ycf15*(2)

Category	Type of genes	Name of genes
	reading frame	

* pseudogenes, number in parentheses represents the quantity of a gene.
