# Supplementary material for: Comparative and phylogenetic analyses of Swertia L. (Gentianaceae) medicinal plants (from Qinghai, China) based on complete chloroplast genomes
Source: Genet Mol Biol. 2021 Dec 13;45(1):e20210092. doi: 10.1590/1678-4685-GMB-2021-0092 (PMC8679245; doi:10.1590/1678-4685-GMB-2021-0092)
Supplement: Table S5 - [file 1415-4757-GMB-45-1-e20210092-s5.pdf]

Genetics and Molecular Biology
https://doi.org/10.1590/1678-4685-GMB-2021-0092

Supplementary Material to "Comparative and phylogenetic analyses of Swertia L. (Gentianaceae) medicinal plants (from Qinghai, China) based on complete chloroplast genomes"

Table S5 - The pseudogenes in *Swertia* chloroplast genomes.

Species	Complete gene loss	Partial gene loss	Premature termination codon	Incomplete copy near boundaries
S. bimaculata MW344293		*rps16*	*infA*	*rps19**; *ycf1*#
S. bimaculata MW344294		*rps16*	*infA*	*rps19**; *ycf1*#
S. bimaculata MW344295		*rps16*	*infA*	*rps19**; *ycf1*#
S. bimaculata MW344296		*rps16*	*infA*	*rps19**; *ycf1*#
S. dichotoma MW344297		*infA*	*rps16*	*rps19**; *ycf1*#
S. dilatata MW344298	*rpl33*	*rps16*; *infA*		*rps19**; *ycf1*#
S. diluta MW338735		*infA*	*rps15*; *rps16*	*rps19**; *ycf1*#
S. erythrosticta MW344299			*rps16*; *infA*	*rps19**; *ycf1*#
S. franchetiana MW344300		*infA*	*rps16*	*rps19**; *ycf1*#
S. franchetiana MW344301		*infA*	*rps16*	*rps19**; *ycf1*#
S. hispidicalyx MH321887	*rpl33*	*rps16*; *infA*		*rps19**; *ycf1*#
S. leducii MN609998			*rps16*; *infA*	*rps19**; *ycf1*#
S. multicaulis MT228730			*rps16*; *infA*	*rps19**; *ycf1*#
S. mussotii KU641021		*infA*	*rps16*	*rps19**; *ycf1*#
S. mussotii MW344302		*infA*	*rps16*	*rps19**; *ycf1*#
S. mussotii MW344303		*infA*	*rps16*	*rps19**; *ycf1*#
S. mussotii MW344304		*infA*	*rps16*	*rps19**; *ycf1*#
S. przewalskii MW344305		*infA*	*rps16*	*rps19**; *ycf1*#
S. souliei MT185926			*rps16*; *infA; ycf1*	*rps19**; *ycf1*#
S. tetraptera MW344306		*infA*	*rps16*	*rps19**; *ycf1*#
S. verticillifolia MF795137		*infA*	*rps16*	*rps19**; *ycf1*#
S. wolfgangiana MW344307		*infA*	*rps16*	*rps19**; *ycf1*#

* means pseudogenes in inverted repeat region a (IRa) close to large single copy region (LSC); # indicates pseudogenes in inverted repeat region b (IRb) close to small single copy region (SSC).
