# Supplementary material for: Comparative and phylogenetic analyses of Swertia L. (Gentianaceae) medicinal plants (from Qinghai, China) based on complete chloroplast genomes
Source: Genet Mol Biol. 2021 Dec 13;45(1):e20210092. doi: 10.1590/1678-4685-GMB-2021-0092 (PMC8679245; doi:10.1590/1678-4685-GMB-2021-0092)
Supplement: Figure S8 - [file 1415-4757-GMB-45-1-e20210092-s14.pdf]

Genetics and Molecular Biology
https://doi.org/10.1590/1678-4685-GMB-2021-0092

Supplementary Material to "Comparative and phylogenetic analyses of Swertia L. (Gentianaceae) medicinal plants (from Qinghai, China) based on complete chloroplast genomes"

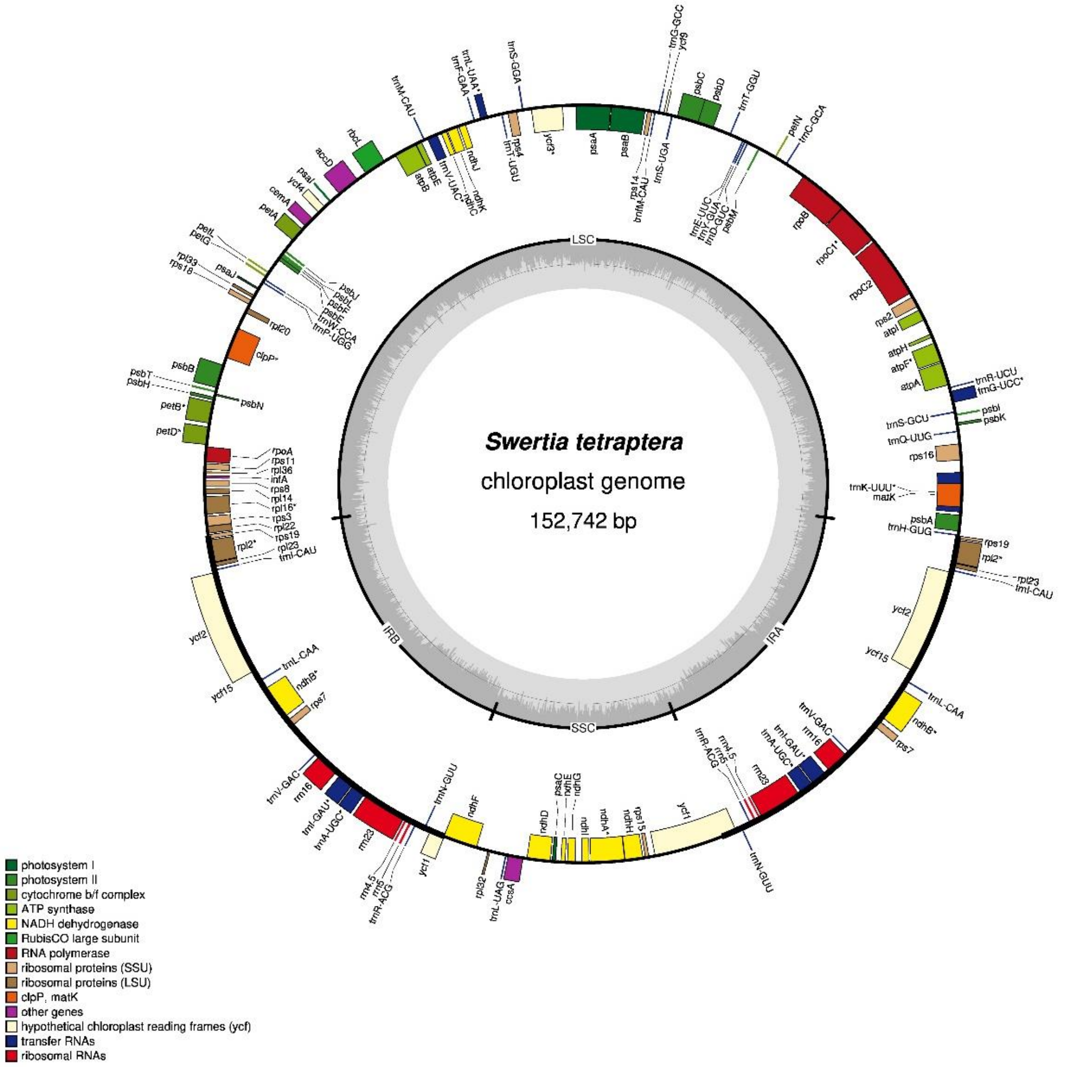

Figure S8 - Gene maps of *S. tetraptera* chloroplast genome. The translation of genes outside the outer circle occurs in a counter-clockwise direction, while the translation of genes inside occurs in a clockwise direction. The dark and light gray colors in the inner circle represent GC and AT content, respectively. Different functional gene groups are highlighted by different colors.
