# Supplementary material for: Comparative and phylogenetic analyses of Swertia L. (Gentianaceae) medicinal plants (from Qinghai, China) based on complete chloroplast genomes
Source: Genet Mol Biol. 2021 Dec 13;45(1):e20210092. doi: 10.1590/1678-4685-GMB-2021-0092 (PMC8679245; doi:10.1590/1678-4685-GMB-2021-0092)
Supplement: Figure S10 - [file 1415-4757-GMB-45-1-e20210092-s16.pdf]

Genetics and Molecular Biology
https://doi.org/10.1590/1678-4685-GMB-2021-0092

Supplementary Material to "Comparative and phylogenetic analyses of Swertia L. (Gentianaceae) medicinal plants (from Qinghai, China) based on complete chloroplast genomes"

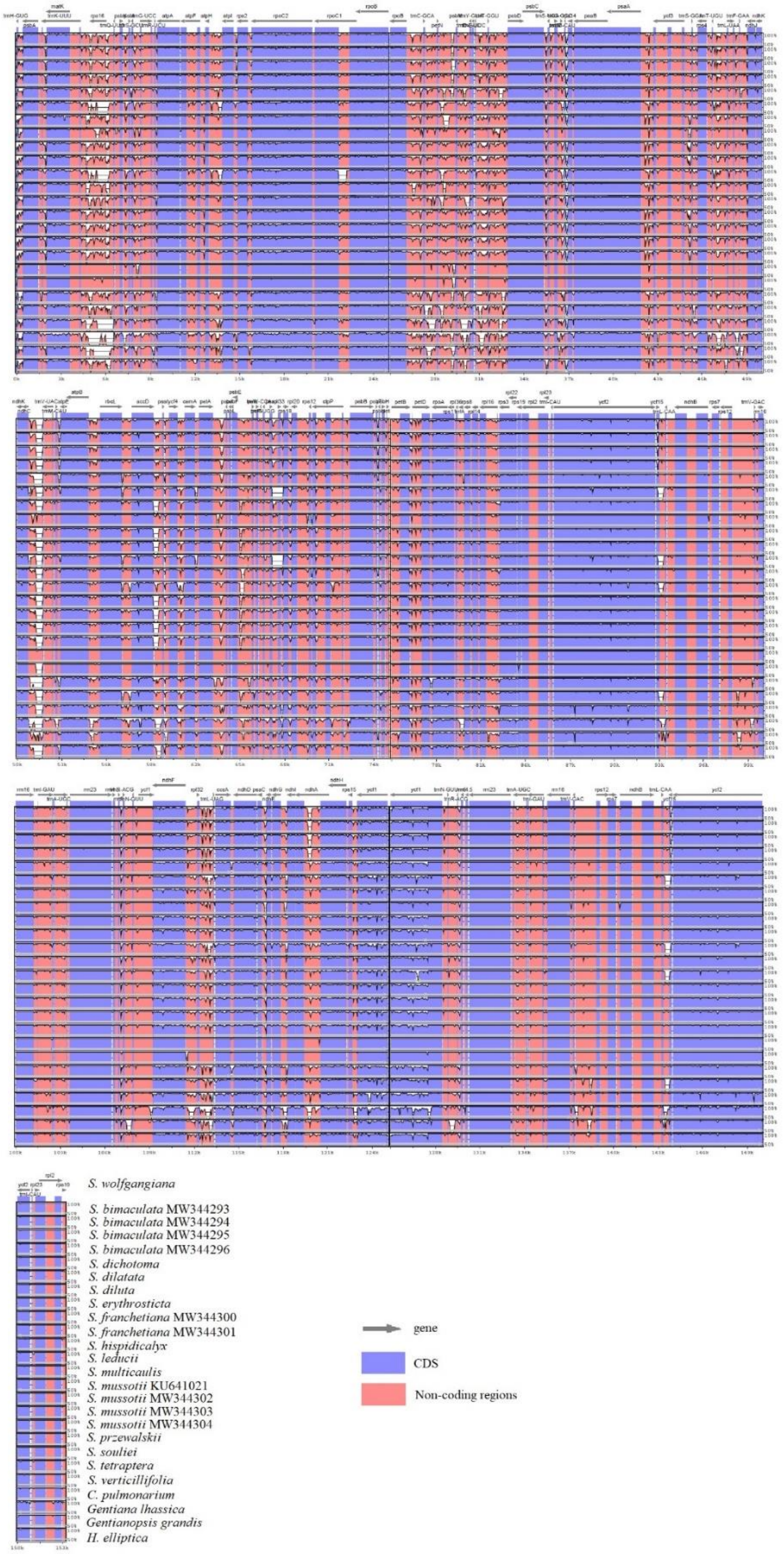

Figure S10 - The alignment of chloroplast genomes for 15 *Swertia* species and four related species. *S. wolfgangiana* was used as a reference. The numbers at the bottom represent the position of the chloroplast genomes, while the percentages on the right represent similarity.
